# Flow Stability of Nanofluid Thin Films on Non-Uniformly Heated Porous Slopes

**DOI:** 10.3390/nano16040247

**Published:** 2026-02-13

**Authors:** Jiawei Li, Xia Li, Liqing Yue, Xinshan Li, Zhaodong Ding

**Affiliations:** 1 School of Mathematical Science, Inner Mongolia University, Hohhot 010021, China; 32336055@mail.imu.edu.cn (J.L.); judy_yue@imu.edu.cn (L.Y.); 2 Inner Mongolia Key Laboratory of Mathematical Modeling and Scientific Computing, Hohhot 010021, China

**Keywords:** nanofluid, thin liquid film, porous medium, Beavers-Joseph slip, hydrodynamic stability

## Abstract

Thin liquid film flows of nanofluids over porous surfaces are central to applications ranging from microfluidic thermal management to precision coating technologies. This study investigates the hydrodynamic and thermal stability of a nanofluid flowing down a non-uniformly heated inclined porous plane subject to the Beavers-Joseph slip boundary condition. Using the long-wave approximation, a nonlinear evolution equation governing the film thickness is derived. The stability characteristics are systematically analyzed via linear stability theory, weakly nonlinear analysis, and fast Fourier transform (FFT) numerical simulations. Quantitative results indicate that the porous medium permeability, density difference, and Marangoni number act as destabilizing factors; specifically, increasing the porous parameter β (from 0 to 0.3), the density ratio $\zeta_{0}$ (from 0 to 5), and the Marangoni number Mn (from 0 to 0.3) significantly reduces the critical Reynolds number and accelerates the onset of interfacial instabilities. In contrast, increasing the nanoparticle volume fraction ϕ from 0 to 0.3 exerts a dominant stabilizing effect by elevating the critical Reynolds number and shrinking the unstable wavenumber domain. Furthermore, nonlinear simulations confirm that higher nanoparticle concentrations effectively suppress the saturation amplitude of disturbances, promoting the eventual stabilization of the liquid film.

## 1. Introduction

Film flow is a ubiquitous phenomenon where a thin, continuous liquid layer moves along a solid surface driven by external forces. It is observed in daily scenarios, such as rainwater sliding on glass [1,2]. In industrial applications, liquid films are pivotal for gas separation, toxic metal recovery, and specifically in the coating industry [3,4]. Crucially, in thermal management systems like microelectronic cooling and thin-film heat exchangers, liquid films serve as efficient agents for heat dissipation and condensation [5,6]. One critical application is liquid film cooling in liquid rocket engines, where coolant is introduced through a porous structure to form a protective film on the combustion chamber wall. Excessive fluctuation of this film can lead to catastrophic engine damage, underscoring the importance of stability analysis.

While the fundamental stability criteria for Newtonian films were established in classical studies [7,8], modern applications often involve complex fluids and multi-physical coupling in porous media. Central to porous wall analysis is the slip boundary condition proposed by Beavers and Joseph [9], later applied by Pascal [10,11] to determine instability conditions for films on porous slopes. However, recent studies demonstrate that non-Newtonian rheology significantly alters flow dynamics in permeable layers. For instance, Mohamad et al. [12] investigated heat and mass transfers on the chemically reactive thermosolutal convective flow of Rivlin–Ericksen fluid over a porous medium, emphasizing the effect of viscous dissipation. Similarly, analytical and numerical simulations were conducted by Yadav et al. [13] to analyze the effects of temperature-reliant thermal conductivity and viscosity disparities on the onset of cellular convective motion in a viscoelastic Oldroyd-B type fluid saturated permeable layer. Furthermore, the onset of Casson fluid convection in a permeable medium layer produced by purely inner heating with a magnetic field was explored by Yadav et al. [14]. Building on these complex interactions, Liu [15] identified three unstable modes (surface, shear, and porous) in film flow, and Hill and Straughan [16] revealed distinct instabilities in fluid-porous layers.

In contrast to classical fluids, nanofluids have garnered attention for their superior thermal conductivity and utility in energy conversion systems [17,18]. While often modeled as homogeneous mixtures, extensive research has established correlations for their thermophysical properties, such as the dependence of dynamic viscosity and effective thermal conductivity on nanoparticle concentration [19,20,21]. Regarding flow dynamics, Lin et al. [22] demonstrated that nanoparticles can suppress linear instability in channel flows, while Zhang et al. [23] analyzed the coupled effects of magnetic fields and velocity slip on power-law nanofluid films. Moreover, thermal convection in a layer of micropolar nanofluid has been investigated by Chand et al. [24] to elucidate the onset of instability and the associated enhancement of heat transfer mechanisms.

Apart from fluid properties, the thermal boundary condition plays a pivotal role in film stability. Since Lin [25] pioneered the study of the Marangoni effect in non-isothermal films, this topic has been extensively explored. Samanta [26,27] investigated film flow on non-uniformly heated inclines, using long-wave perturbation to derive nonlinear surface wave equations that capture both linear and sideband instabilities. Further expanding on complex scenarios, Kirkinis and Andreev [28] examined the stabilizing role of odd viscosity, while Mukhopadhyay et al. [29] revealed that the Marangoni effect destabilizes flow over wavy bottoms, with bottom steepness exhibiting a dual effect on linear stability.

However, existing literature rarely considers the simultaneous interaction of nanofluid rheology, non-uniform heating, and porous substrates under slip conditions. This paper addresses this gap by deriving a nonlinear evolution equation for nanofluid film thickness, incorporating the Beavers-Joseph slip boundary condition and systematic asymptotic expansion. The novelty of this work lies in the integrated analysis of how nanoparticle volume fraction and density difference couple with the slip boundary to alter flow stability. By employing linear stability analysis, weakly nonlinear theory, and numerical simulation, we clarify these complex mechanisms. These findings provide theoretical guidance for optimizing thermal management in micro-electromechanical systems (MEMS) and controlling coating uniformity in precision manufacturing.

## 2. Mathematical Model

**Figure 1:**
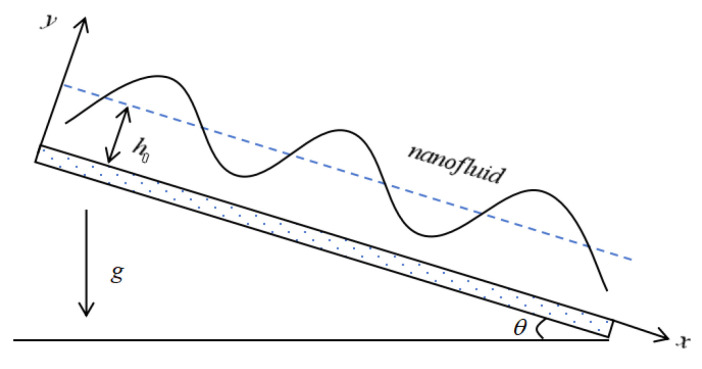
Schematic diagram of the flow of nanofluids along a porous inclined plane.

We have conducted a study on the flow of incompressible viscous nanofluids under the drive of gravity within a two-dimensional space. The nanofluids flow downward along an inclined slip plane with non-uniform heating. As depicted in Figure 1, a Cartesian coordinate system (x,y) has been introduced. In this system, the *x*-axis coincides with the bottom of the plane and is oriented in the direction of the flow, while the *y*-axis is perpendicular to the inclined plane and points upwards towards the interior of the liquid. The inclination angle of the bottom plate is denoted as θ, and the acceleration resulting from gravity is indicated by *g*. The position of the free surface of the liquid film is expressed as y=h(x,t), where $h_{0}$ stands for the thickness of the undisturbed film.

Within the (x,y) coordinate system, the velocity field is defined as u=(u,v), with *u* and *v* denoting the flow velocities in the horizontal and vertical directions respectively. The dynamic characteristics of incompressible fluids can be depicted by means of the continuity equation and the momentum equation.

(1) $$\frac{\partial u}{\partial x}+\frac{\partial v}{\partial y}=0,$$

(2) $$\rho_{f}\left(\frac{\partial u}{\partial t}+u\frac{\partial u}{\partial x}+v\frac{\partial u}{\partial y}\right)=-\frac{\partial p}{\partial x}+\rho_{n}g\sin\theta +\mu_{n}\left(\frac{\partial^{2}u}{\partial x^{2}}+\frac{\partial^{2}u}{\partial y^{2}}\right),$$

(3) $$\rho_{f}\left(\frac{\partial v}{\partial t}+u\frac{\partial v}{\partial x}+v\frac{\partial v}{\partial y}\right)=-\frac{\partial p}{\partial y}-\rho_{n}g\cos\theta +\mu_{n}\left(\frac{\partial^{2}v}{\partial x^{2}}+\frac{\partial^{2}v}{\partial y^{2}}\right),$$

(4) $$\frac{\partial T}{\partial t}+u\frac{\partial T}{\partial x}+v\frac{\partial T}{\partial y}=k_{n}\left(\frac{\partial^{2}T}{\partial x^{2}}+\frac{\partial^{2}T}{\partial y^{2}}\right),$$

among these parameters, $\rho_{f}$ and $\rho_{n}$ denote the densities of the base fluid and the nanofluid respectively. *p* refers to the liquid pressure, $\mu_{n}$ stands for the viscosity coefficient of the nanofluid, *T* indicates the temperature, and $k_{n}$ is the thermal diffusivity coefficient of the nanofluid, which is supposed to be a constant.

In this framework, the nanofluid is treated as a single-phase homogeneous fluid with effective thermophysical properties. While this approach is a standard first approximation in thin-film stability analysis, we acknowledge its inherent limitations. In actual nanofluid systems, effects such as nanoparticle migration, thermophoresis, and Brownian diffusion can be significant under strong thermal gradients or high shear rates. However, for the stable, well-dispersed dispersions at moderate volume fractions considered in the present study, these secondary slip mechanisms are assumed to be negligible compared to the dominant Marangoni and capillary forces. This simplification allows for a focused investigation on the fundamental interfacial dynamics while accounting for the enhanced transport properties of the nanofluid.

The thin film employs the Beavers-Joseph slip boundary condition at the location where the inclined bottom plate has y=0. The applicable condition with respect to the tangential velocity at the interface is

(5) $${\left.\frac{\partial u}{\partial y}\right|}_{y=0}=\frac{\alpha }{\sqrt{k_{p}}}\left(u-u_{p}\right),$$

where $k_{p}$ stands for the permeability of the porous medium, α is a dimensionless parameter relying on the structure of the porous medium, and $u_{p}$ represents the Darcy’s average flow velocity in the *x* direction. Given that the normal velocity within the boundary layer stays constant, the condition at the interface is

(6) $${\left.v\right|}_{y=0}=v_{p},$$

where $v_{p}$ represents the Darcy’s average flow velocity in the *y* direction.

When the characteristic length of the pores in the porous medium is significantly smaller than the thickness of the liquid film, the wall condition is simplified through the application of Pascal’s scale analysis as

(7) $$\left\{\begin{array}{c} \partial u/\partial y=(\alpha /\sqrt{k_{p}})u,\\ v=0, \end{array}\;y=0.\right.$$

This simplification captures the kinematic effect of the porous substrate via the interface slip relationship, while the secondary internal Darcy dynamics are neglected following Pascal’s scale analysis to focus on the film’s interfacial evolution [10,11].

At the substrate with y=0, the boundary condition regarding the temperature is

(8) $$T=T_{g}+bx,$$

where $T_{g}$ denotes the ambient temperature. The parameter $b=\Delta T/\hat{\lambda }$ represents the linear rate of temperature variation, where $\Delta T=T_{H}-T_{C}$. Here, $T_{H}$ and $T_{C}$ refer to the temperatures of the high temperature and low temperature regions of the substrate respectively.

At the free surface with y=h(x,t), the dynamic and kinematic boundary conditions [29] are given respectively as (The detailed derivation of these equations can be found in Appendix A).

(9) $$\frac{1}{\sqrt{1+h_{x}^{2}}}\left[\mu_{n}\left(1-h_{x}^{2}\right)\left(\frac{\partial v}{\partial x}+\frac{\partial u}{\partial y}\right)+2\mu_{n}\left(\frac{\partial v}{\partial y}-\frac{\partial u}{\partial x}\right)h_{x}\right]=\frac{\partial \gamma }{\partial x}+h_{x}\frac{\partial \gamma }{\partial y},$$

(10) $$p_{g}-p+\frac{\mu_{n}}{1+h_{x}^{2}}\left[2\frac{\partial v}{\partial y}\left(1-h_{x}^{2}\right)-2h_{x}\left(\frac{\partial u}{\partial y}+\frac{\partial v}{\partial x}\right)\right]=\frac{\gamma h_{xx}}{{\left(1+h_{x}^{2}\right)}^{3/2}},$$

(11) $$v=\frac{\partial h}{\partial t}+u\frac{\partial h}{\partial x},$$

here, γ denotes the surface tension, and it is postulated that γ varies linearly within a narrow temperature difference range

(12) $$\gamma =\gamma_{0}-\sigma \left(T-T_{g}\right),$$

where $\gamma_{0}$ represents the surface tension at the reference temperature $T_{g}$, and σ is defined as ${\sigma =-\partial \gamma /\partial T}_{T=T_{g}}$. σ is the coefficient of thermal surface tension.

The thermal balance at the deformable free surface y=h(x,t) is governed by Newton’s law of cooling, which characterizes the convective heat exchange between the liquid film and the ambient environment [29]. Specifically, Equation (13) defines a Robin-type boundary condition:

(13) $$\begin{array}{c} -\frac{\lambda }{\sqrt{1+h_{x}^{2}}}\left[-\frac{h_{x}}{{\left(1+h\right)}^{2}}\frac{\partial T}{\partial x}+\frac{\partial T}{\partial y}\right]=k_{g}\left(T-T_{g}\right), \end{array}$$

where λ denotes the thermal conductivity coefficient and $k_{g}$ represents the heat transfer coefficient between the fluid and the air.

The following dimensionless parameters are introduced to nondimensionalize the equations (indicated by the superscript asterisk)

(14) $$\begin{array}{cc} \; & x^{*}=\frac{x}{\hat{\lambda }},\;y^{*}=\frac{y}{h_{0}},\;h^{*}=\frac{h}{h_{0}},\;u^{*}=\frac{u}{u_{0}},\;v^{*}=\frac{\hat{\lambda }v}{h_{0}u_{0}},\;\\ \; & t^{*}=\frac{u_{0}t}{\hat{\lambda }},\;p^{*}=\frac{p}{\mu_{f}u_{0}/h_{0}},\;T^{*}=\frac{T-T_{g}}{\Delta T},\;\phi^{*}=\frac{\phi }{\phi_{ave}}, \end{array}$$

where $u_{0}=\rho_{f}gh_{0}^{2}\sin\theta /3\mu_{f}$ represents the Nusselt velocity. $h_{0}$ denotes the undisturbed film thickness as well as the transverse length. $\hat{\lambda }$ is the characteristic longitudinal length scale, being significantly longer than the film thickness. $\epsilon =h_{0}/\hat{\lambda }\ll 1$ is defined as the aspect ratio, and $\phi_{ave}$ is the average volume fraction of nanoparticles over a certain time interval.

By substituting the dimensionless parameters (14) into the governing equations and boundary conditions and removing the asterisks, we obtain

(15) $$\frac{\partial u}{\partial x}+\frac{\partial v}{\partial y}=0,$$

(16) $$\epsilon Re\left(\frac{\partial u}{\partial t}+u\frac{\partial u}{\partial x}+v\frac{\partial u}{\partial y}\right)=-\epsilon \frac{\partial p}{\partial x}+3F_{\rho }+F_{\mu }\left(\epsilon^{2}\frac{\partial^{2}u}{\partial x^{2}}+\frac{\partial^{2}u}{\partial y^{2}}\right),$$

(17) $$\epsilon^{2}Re\left(\frac{\partial v}{\partial t}+u\frac{\partial v}{\partial x}+v\frac{\partial v}{\partial y}\right)=-\frac{\partial p}{\partial y}-3F_{\rho }\cot\theta +F_{\mu }\left(\epsilon^{3}\frac{\partial^{2}v}{\partial x^{2}}+\epsilon \frac{\partial^{2}v}{\partial y^{2}}\right),$$

(18) $$\epsilon RePr\left(\frac{\partial T}{\partial t}+u\frac{\partial T}{\partial x}+v\frac{\partial T}{\partial y}\right)=\epsilon^{2}\frac{\partial^{2}T}{\partial x^{2}}+\frac{\partial^{2}T}{\partial y^{2}}.$$

where the dimensionless parameters are defined as the Reynolds number Re, the Prandtl number Pr, the relative density $F_{\rho }$, and the relative viscosity $F_{\mu }$:

$$Re=\frac{\rho_{f}u_{0}h_{0}}{\mu_{f}},Pr=\frac{\mu_{f}}{\rho_{f}k_{nf}},F_{\rho }=\frac{\rho_{n}}{\rho_{f}},F_{\mu }=\frac{\mu_{n}}{\mu_{f}}.$$

The Beavers-Joseph slip boundary condition of the thin film on the interface is expressed as

(19) $$\left\{\begin{array}{c} \partial u/\partial y=u/\beta ,\\ v=0, \end{array}\;y=0,\right.$$

where $\beta =\sqrt{k_{p}}/\alpha h$ is used to denote the dimensionless parameter for the permeability of the porous medium.

The thermal boundary condition at the substrate (y=0) is simplified as:

(20) T=x.

At the free surface where y=h(x,t)

(21) $$\frac{1}{\sqrt{1+\epsilon^{2}h_{x}^{2}}}\left[F_{\mu }\left(1-\epsilon^{2}h_{x}^{2}\right)\left(\epsilon^{2}\frac{\partial v}{\partial x}+\frac{\partial u}{\partial y}\right)+2\epsilon^{2}F_{\mu }h_{x}\left(\frac{\partial v}{\partial y}-\frac{\partial u}{\partial x}\right)\right]=-Mn\left(\frac{\partial T}{\partial x}+h_{x}\frac{\partial T}{\partial y}\right),$$

(22) $${\bar{p}}_{g}-p+\frac{F_{\mu }}{1+\epsilon^{2}h_{x}^{2}}\left[2\epsilon \frac{\partial v}{\partial y}\left(1-\epsilon^{2}h_{x}^{2}\right)-2\epsilon h_{x}\left(\frac{\partial u}{\partial y}+\epsilon^{2}\frac{\partial v}{\partial x}\right)\right]=\frac{h_{xx}}{Ca^{\prime }{\left(1+\epsilon^{2}h_{x}^{2}\right)}^{3/2}},$$

(23) $$v=\frac{\partial h}{\partial t}+u\frac{\partial h}{\partial x},$$

(24) $$-\frac{1}{\sqrt{1+\epsilon^{2}h_{x}^{2}}}\left[-\frac{\epsilon^{2}h_{x}}{{\left(1+hh_{0}\right)}^{2}}\frac{\partial T}{\partial x}+\frac{\partial T}{\partial y}\right]=B_{i}T,$$

where ${\bar{p}}_{g}=p_{g}/\left(\mu_{f}u_{0}/h_{0}\right)$, and the dimensionless parameters are the Marangoni number Mn, the capillary number Ca and the Biot number Bi, whose forms are respectively

$$Mn=\frac{\epsilon \gamma \Delta T}{\mu_{f}u_{0}},Ca^{\prime }=\frac{Ca}{\epsilon^{2}}=\frac{\mu_{f}u_{0}}{\epsilon^{2}\gamma },Bi=\frac{k_{g}h_{0}}{\lambda }.$$

Expand the physical quantities *u*, *v*, *p* and *T* into the form of power series of the small parameter ϵ

(25) $$\begin{array}{cc} u & =u_{0}+\epsilon u_{1}+\cdots ,\\ v & =v_{0}+\epsilon v_{1}+\cdots ,\\ p & =p_{0}+\epsilon p_{1}+\cdots ,\\ T & =T_{0}+\epsilon T_{1}+\cdots . \end{array}$$

Substitute the asymptotic expansion (25) into the dimensionless equations, ignoring the O(δ) and higher-order terms, and the resulting zeroth order governing equation is given by

(26) $$\frac{\partial u_{0}}{\partial x}+\frac{\partial v_{0}}{\partial y}=0,$$

(27) $$3F_{\rho }+F_{\mu }\frac{\partial^{2}u_{0}}{\partial y^{2}}=0,$$

(28) $$-\frac{\partial p_{0}}{\partial y}-3F_{\rho }\cot\theta =0,$$

(29) $$\frac{\partial^{2}T_{0}}{\partial y^{2}}=0.$$

The boundary conditions at the zeroth order are as follows

(30) $${\left.\frac{\partial u_{0}}{\partial y}\right|}_{y=0}=\frac{1}{\beta }u_{0},$$

(31) $${v_{0}|}_{y=0}=0,$$

(32) $${T_{0}|}_{y=0}=x,$$

(33) $${\left.F_{\mu }\frac{\partial u_{0}}{\partial y}\right|}_{y=h}=-Mn\left(\frac{\partial T_{0}}{\partial x}+\frac{\partial h}{\partial x}\frac{\partial T_{0}}{\partial y}\right),$$

(34) $$\left({\bar{p}}_{g}-p_{0}+F_{\mu }\right){|}_{y=h}=\frac{1}{Ca^{\prime }}\frac{\partial^{2}h}{\partial x^{2}},$$

(35) $$v_{0}{|}_{y=h}=\frac{\partial h}{\partial t}+u_{0}\frac{\partial h}{\partial x},$$

(36) $${\left.\frac{\partial T_{0}}{\partial y}\right|}_{y=h}=0.$$

By solving the system of equations, we obtain

(37) $$u_{0}=-\frac{3F_{\rho }}{2F_{\mu }}y^{2}+\frac{3F_{\rho }}{F_{\mu }}hy+\frac{3F_{\rho }}{F_{\mu }}\beta h-\frac{Mn}{F_{\mu }}y-\frac{Mn}{F_{\mu }}\beta ,$$

(38) $$v_{0}=-\frac{3F_{\rho }}{2F_{\mu }}\frac{\partial h}{\partial x}y^{2}-\frac{3F_{\rho }}{F_{\mu }}\frac{\partial h}{\partial x}\beta y,$$

(39) $$p_{0}=-3F_{\rho }\cot\theta \left(y-h\right)+{\bar{p}}_{g}+F_{\mu }-\frac{1}{C_{a}^{\prime }}\frac{\partial^{2}h}{\partial x^{2}}.$$

(40) $$T_{0}=x.$$

This result arises from the assumption that the Biot number is of order $O(\epsilon^{2})$ (i.e., Bi≪1), representing a regime where heat transfer to the ambient gas is negligible compared to the streamwise thermal transport. This scaling argument is consistent with the established framework for non-isothermal film flows on linearly heated substrates, as detailed by Mukhopadhyay and Haldar [30].

Similar to the derivation of the zero-order equation, we substitute the asymptotic expansion into the dimensionless equations again, and thus the first-order governing equation is given by

(41) $$\frac{\partial u_{1}}{\partial x}+\frac{\partial v_{1}}{\partial y}=0,$$

(42) $$Re\left(\frac{\partial u_{0}}{\partial t}+u_{0}\frac{\partial u_{0}}{\partial x}+v_{0}\frac{\partial u_{0}}{\partial y}\right)=-\frac{\partial p_{0}}{\partial x}+F_{\mu }\frac{\partial^{2}u_{1}}{\partial y^{2}},$$

(43) $$0=-\frac{\partial p_{1}}{\partial y}+F_{\mu }\frac{\partial^{2}v_{0}}{\partial y^{2}},$$

(44) $$RePr\left(\frac{\partial T_{0}}{\partial t}+u_{0}\frac{\partial T_{0}}{\partial x}+v_{0}\frac{\partial T_{0}}{\partial y}\right)=\frac{\partial^{2}T_{1}}{\partial y^{2}}.$$

The boundary conditions at the first order are as follows

(45) $${v_{1}|}_{y=0}=0,$$

(46) $${\left.\frac{\partial u_{1}}{\partial y}\right|}_{y=0}=\frac{1}{\beta }u_{1},$$

(47) $$F_{\mu }\frac{\partial u_{1}}{\partial y}{|}_{y=h}=-Mn\left(\frac{\partial T_{1}}{\partial x}+\frac{\partial h}{\partial x}\frac{\partial T_{1}}{\partial y}\right),$$

(48) $${\left.\left(-p_{1}+2F_{\mu }\left(\frac{\partial v_{0}}{\partial y}-\frac{\partial h}{\partial x}\frac{\partial u_{0}}{\partial y}\right)\right)\right|}_{y=h}=0,$$

(49) $$v_{1}{|}_{y=h}=u_{1}\frac{\partial h}{\partial x},$$

(50) $$\frac{\partial T_{1}}{\partial y}{|}_{y=h}=0.$$

By solving the first-order equation, the expression for $u_{1}$ is obtained as

(51) $$\begin{array}{cc} u_{1}= & Re(\frac{F_{\rho }}{2F_{\mu }^{2}}\frac{\partial h}{\partial t}\left[y^{3}+3\beta y^{2}-3h\left(y+\beta \right)\left(h+2\beta \right)\right]\\ \; & +\frac{3F_{\rho }^{2}}{8F_{\mu }^{3}}\frac{\partial h}{\partial x}\left[\left(h+\beta \right)y^{4}+4\beta hy^{2}\left(y+3\beta \right)-4h^{2}\left(y+\beta \right)\left(h^{2}+4\beta h+6\beta^{2}\right)\right]\\ \; & -\frac{F_{\rho }}{8F_{\mu }^{3}}Mn\frac{\partial h}{\partial x}\left[y^{4}+4\beta y^{2}\left(y+3\beta \right)-4h\left(y+\beta \right)\left(h^{2}+3\beta h+3\beta^{2}\right)\right])\\ \; & -MnRePr\frac{\partial h}{\partial x}(\frac{F_{\rho }}{4F_{\mu }^{2}}\left[y^{4}+2y^{2}\left(y-h\right)\left(2h+3\beta \right)-4h^{2}\left(y+\beta \right)\left(2+3\beta \right)\right]\\ \; & +\frac{Mn}{6F_{\mu }^{2}}\left[-y^{3}+3hy^{2}+3h\left(y+\beta \right)\left(h+2\beta \right)\right])\\ \; & +\left(\frac{3F_{\rho }}{2F_{\mu }}\frac{\partial h}{\partial x}\cot\theta -\frac{1}{2Ca^{\prime }F_{\mu }}\frac{\partial^{3}h}{\partial x^{3}}\right)\left(y^{2}-2hy-2\beta y\right) \end{array}$$

Define the local flow rate q(x,t) as

(52) $$q\left(x,t\right)=\int_{0}^{h\left(x,t\right)}u\left(x,y,t\right)dy,$$

where $u\left(x,y,t\right)=u_{0}\left(x,y,t\right)+\epsilon u_{1}\left(x,y,t\right)+O\left(\epsilon^{2}\right)$. Substitute *u* into Equation (52) and solve the integral to obtain

(53) $$\begin{array}{cc} q\left(x,t\right)= & \frac{F_{\rho }}{F_{\mu }}h^{3}+\frac{3F_{\rho }}{F_{\mu }}\beta h^{2}-\frac{Mn}{2F_{\mu }}h^{2}-\frac{Mn}{F_{\mu }}\beta h+\epsilon [\frac{6F_{\rho }^{2}}{5F_{\mu }^{3}}\frac{\partial h}{\partial x}Reh^{6}+\frac{36F_{\rho }^{2}}{5F_{\mu }^{3}}\frac{\partial h}{\partial x}Re\beta h^{5}-\frac{2F_{\rho }}{5F_{\mu }^{3}}\frac{\partial h}{\partial x}\\ \; & \times \;MnReh^{5}-\frac{2F_{\rho }}{F_{\mu }^{3}}\frac{\partial h}{\partial x}MnRe\beta h^{4}+\frac{45F_{\rho }^{2}}{2F_{\mu }^{3}}\frac{\partial h}{\partial x}Re\beta^{2}h^{4}-\frac{5F_{\rho }}{2F_{\mu }^{3}}\frac{\partial h}{\partial x}MnRe\beta^{2}h^{3}+\frac{9F_{\rho }^{2}}{F_{\mu }^{3}}\frac{\partial h}{\partial x}\\ \; & \times \;Re\beta^{3}h^{3}-\frac{5F_{\rho }}{2F_{\mu }^{3}}\frac{\partial h}{\partial x}Re\beta^{2}h^{3}+\frac{3F_{\rho }}{2F_{\mu }^{2}}\frac{\partial h}{\partial x}MnRe\beta^{2}h^{2}-\frac{F_{\rho }}{F_{\mu }}\frac{\partial h}{\partial x}\cot\theta h^{3}+\frac{1}{3Ca^{\prime }F_{\mu }}\frac{\partial^{3}h}{\partial x^{3}}h^{3}\\ \; & -\;\frac{3F_{\rho }}{F_{\mu }}\frac{\partial h}{\partial x}\cot\theta \beta h^{2}+\frac{1}{Ca^{\prime }F_{\mu }}\frac{\partial^{3}h}{\partial x^{3}}\beta h^{2}-MnRePr(-\frac{F_{\rho }}{30F_{\mu }^{2}}\frac{\partial h}{\partial x}h^{5}-\frac{13F_{\rho }}{8F_{\mu }^{2}}\frac{\partial h}{\partial x}\beta h^{4}+\frac{3Mn}{8F_{\mu }^{2}}\\ \; & \times \;\frac{\partial h}{\partial x}h^{4}+\frac{4Mn}{3F_{\mu }^{2}}\frac{\partial h}{\partial x}\beta h^{3}-\frac{4F_{\rho }}{F_{\mu }^{2}}\frac{\partial h}{\partial x}\beta^{2}h^{3}-\frac{F_{\rho }}{F_{\mu }^{2}}\frac{\partial h}{\partial x}h^{4}-\frac{2F_{\rho }}{F_{\mu }^{2}}\frac{\partial h}{\partial x}\beta h^{3}+\frac{Mn}{F_{\mu }^{2}}\frac{\partial h}{\partial x}\beta^{2}h^{2})]. \end{array}$$

Employing an alternative form of the kinematic boundary condition

(54) $$\frac{\partial h}{\partial t}+\frac{\partial q}{\partial x}=0.$$

We derive the nonlinear evolution equation for the film thickness

(55) $$\begin{array}{cc} \; & \frac{\partial h}{\partial t}+\frac{\partial }{\partial x}[\frac{F_{\rho }}{F_{\mu }}h^{3}+\frac{3F_{\rho }}{F_{\mu }}\beta h^{2}-\frac{Mn}{2F_{\mu }}h^{2}-\frac{Mn}{F_{\mu }}\beta h+\frac{6F_{\rho }^{2}}{5F_{\mu }^{3}}\frac{\partial h}{\partial x}Reh^{6}+\frac{36F_{\rho }^{2}}{5F_{\mu }^{3}}\frac{\partial h}{\partial x}Re\beta h^{5}-\frac{2F_{\rho }}{5F_{\mu }^{3}}\frac{\partial h}{\partial x}\\ \; & \times \;MnReh^{5}-\frac{2F_{\rho }}{F_{\mu }^{3}}\frac{\partial h}{\partial x}MnRe\beta h^{4}+\frac{45F_{\rho }^{2}}{2F_{\mu }^{3}}\frac{\partial h}{\partial x}Re\beta^{2}h^{4}-\frac{5F_{\rho }}{2F_{\mu }^{3}}\frac{\partial h}{\partial x}MnRe\beta^{2}h^{3}+\frac{9F_{\rho }^{2}}{F_{\mu }^{3}}\frac{\partial h}{\partial x}\\ \; & \times \;Re\beta^{3}h^{3}-\frac{5F_{\rho }}{2F_{\mu }^{3}}\frac{\partial h}{\partial x}Re\beta^{2}h^{3}+\frac{3F_{\rho }}{2F_{\mu }^{2}}\frac{\partial h}{\partial x}MnRe\beta^{2}h^{2}-\frac{F_{\rho }}{F_{\mu }}\frac{\partial h}{\partial x}\cot\theta h^{3}+\frac{1}{3Ca^{\prime }F_{\mu }}\frac{\partial^{3}h}{\partial x^{3}}h^{3}\\ \; & -\;\frac{3F_{\rho }}{F_{\mu }}\frac{\partial h}{\partial x}\cot\theta \beta h^{2}+\frac{1}{Ca^{\prime }F_{\mu }}\frac{\partial^{3}h}{\partial x^{3}}\beta h^{2}-MnRePr(-\frac{F_{\rho }}{30F_{\mu }^{2}}\frac{\partial h}{\partial x}h^{5}-\frac{13F_{\rho }}{8F_{\mu }^{2}}\frac{\partial h}{\partial x}\beta h^{4}+\frac{3Mn}{8F_{\mu }^{2}}\\ \; & \times \;\frac{\partial h}{\partial x}h^{4}+\frac{4Mn}{3F_{\mu }^{2}}\frac{\partial h}{\partial x}\beta h^{3}-\frac{4F_{\rho }}{F_{\mu }^{2}}\frac{\partial h}{\partial x}\beta^{2}h^{3}-\frac{F_{\rho }}{F_{\mu }^{2}}\frac{\partial h}{\partial x}h^{4}-\frac{2F_{\rho }}{F_{\mu }^{2}}\frac{\partial h}{\partial x}\beta h^{3}+\frac{Mn}{F_{\mu }^{2}}\frac{\partial h}{\partial x}\beta^{2}h^{2})]=0. \end{array}$$

To validate the derivation of the nonlinear evolution equation, we consider the limiting case of a pure Newtonian fluid flowing down a solid, isothermal inclined plane. By setting the nanoparticle volume fraction ϕ=0 (yielding $F_{\rho }=F_{\mu }=1$), the porous permeability parameter β=0, and the Marangoni number Mn=0, Equation (55) strictly reduces to the classical Benney equation describing the evolution of a thin liquid film [31,32]. This consistency confirms that the present model correctly captures the fundamental nonlinear hydrodynamics in the Newtonian limit.

## 3. Linear Stability Analysis

For the investigation of the stability of film flow, the film thickness under the perturbed state can be expressed as $h\left(x,t\right)=1+h_{1}\left(x,t\right)$, where $|h_{1}|\ll 1$ denotes the dimensionless unperturbed film thickness. Upon substituting it into Equation (54) and neglecting the higher-order terms of $O(h_{1})$ and retaining up to $O(h_{1}^{3})$, we obtain

(56) $$\begin{array}{cc} \; & \frac{\partial h_{1}}{\partial t}+A\frac{\partial h_{1}}{\partial x}+B\frac{\partial^{2}h_{1}}{\partial x^{2}}+C\frac{\partial^{4}h_{1}}{\partial x^{4}}+A^{\prime }h_{1}\frac{\partial h_{1}}{\partial x}+B^{\prime }\left[h_{1}\frac{\partial^{2}h_{1}}{\partial x^{2}}+{\left(\frac{\partial h_{1}}{\partial x}\right)}^{2}\right]+C^{\prime }(h_{1}\frac{\partial^{4}h_{1}}{\partial x^{4}}+\frac{\partial h_{1}}{\partial x}\\ \; & \times \frac{\partial^{3}h_{1}}{\partial x^{3}})+\frac{1}{2}A^{\prime \prime }h_{1}^{2}\frac{\partial h_{1}}{\partial x}+B^{\prime \prime }\left[\frac{1}{2}h_{1}^{2}\frac{\partial^{2}h_{1}}{\partial x^{2}}+h_{1}{\left(\frac{\partial h_{1}}{\partial x}\right)}^{2}\right]+C^{\prime \prime }\left[\frac{1}{2}h_{1}^{2}\frac{\partial^{4}h_{1}}{\partial x^{4}}+h_{1}\frac{\partial h_{1}}{\partial x}\frac{\partial^{3}h_{1}}{\partial x^{3}}\right]=0, \end{array}$$

where

(57) $$\begin{array}{cc} A\left(h\right)= & \frac{3F_{\rho }}{F_{\mu }}h^{2}+\left(\frac{6F_{\rho }}{F_{\mu }}\beta -\frac{Mn}{F_{\mu }}\right)h-\frac{Mn}{F_{\mu }}\beta ,\\ B\left(h\right)= & \frac{6F_{\rho }^{2}}{5F_{\mu }^{3}}h^{6}Re+\left(\frac{36F_{\rho }^{2}}{5F_{\mu }^{3}}Re\beta -\frac{F_{\rho }}{5F_{\mu }^{3}}MnRe+\frac{F_{\rho }}{30F_{\mu }^{2}}MnRePr\right)h^{5}\\ \; & +\left(-\frac{2F_{\rho }}{F_{\mu }^{3}}MnRe\beta +\frac{45F_{\rho }^{2}}{2F_{\mu }^{3}}Re\beta^{2}+\frac{13F_{\rho }}{8F_{\mu }^{2}}\beta MnRePr-\frac{3Mn^{2}}{8F_{\mu }^{2}}RePr+\frac{F_{\rho }}{F_{\mu }^{2}}MnRePr\right)h^{4}\\ \; & +(\frac{5F_{\rho }}{2F_{\mu }^{3}}MnRe\beta^{2}+\frac{9F_{\rho }^{2}}{F_{\mu }^{3}}Re\beta^{3}-\frac{F_{\rho }}{2F_{\mu }^{3}}Re\beta^{2}-\frac{F_{\rho }}{F_{\mu }}\cot\theta -\frac{4Mn^{2}}{3F_{\mu }^{2}}\beta RePr+\frac{4F_{\rho }}{F_{\mu }^{2}}\beta^{2}MnRePr\\ \; & +\frac{2F_{\rho }}{F_{\mu }^{2}}\beta MnRePr)h^{3}+\left(\frac{3F_{\rho }}{2F_{\mu }^{2}}MnRe\beta^{2}-\frac{3F_{\rho }}{F_{\mu }}\cot\theta \beta -\frac{Mn^{2}}{F_{\mu }^{2}}\beta^{2}RePr\right)h^{2},\\ C\left(h\right)= & \frac{1}{3Ca^{\prime }F_{\mu }}h^{3}+\frac{\beta }{Ca^{\prime }F_{\mu }}h^{2}, \end{array}$$

among them, *A*, *B*, and *C* as well as their corresponding derivatives denote the values when h=1.

The physical property parameters of nanofluids are provided by the subsequent formula, where ϕ denotes the nanoparticle volume fraction.

(58) $$F_{\mu }=\frac{\mu_{n}}{\mu_{f}}={\left(1-\phi_{ave}\phi \right)}^{-2.5},\;F_{\rho }=\frac{\rho_{n}}{\rho_{f}}=1+\zeta_{0}\phi_{ave}\phi ,$$

in this paper, the formulas for the relative viscosity and relative density of nanofluids are estimated values derived from theoretical models, which are used to characterize the physical properties of nanofluids under the conditions of this study. Specifically, $F_{\mu }$ adopts the formula extended by Brinkman [33], which is based on the well-known Einstein equation [34]. Meanwhile, $F_{\rho }$ has been proven to be appropriate through the experiments of Pak and Cho [35] and is widely accepted. The coefficient $\zeta_{0}$ represents the normal density difference between nanoparticles and the base fluid. Since most types of nanoparticles are heavier than common base fluids, in this study, the value of $\zeta_{0}$ is considered to be in the range of 0 to 5.

By assuming that the perturbation presents in the form of a sine wave, the linear response of the thin film was studied, that is

(59) $$h_{1}=\Gamma \left[\exp\left\{i\left(kx-\omega t\right)\right\}\right]+c.c.,$$

where Γ≪1 is the amplitude of the perturbation, and c.c. represents the complex conjugate. The real number *k* denotes the wave number, and $\omega =\omega_{r}+i\omega_{i}$ is the complex frequency. Substituting Equation (59) into Equation (56) and considering its linear part, we obtain the dispersion relation in the sinusoidal mode as

(60) $$D_{1}\left(\omega ,k\right)=-i\omega +Aik-Bk^{2}+Ck^{4}=0.$$

In Equation (60), the real part and the imaginary part of ω are respectively expressed as

(61) $$\begin{array}{cc} \; & \omega_{r}=Ak,\\ \; & \omega_{i}=Bk^{2}-Ck^{4}, \end{array}$$

where $\omega_{r}$ is the oscillation frequency, and $\omega_{i}$ is the linear growth rate of the amplitude. If the linear growth rate of the amplitude $\omega_{i}>0$, the flow is linearly unstable; on the contrary, the flow is stable. If $\omega_{i}=0$, the flow is said to be neutrally stable, and the critical Reynolds number at this time can be obtained as

(62) $$\begin{array}{c} Re_{c}=\frac{\frac{F_{\mu }^{2}}{F_{\rho }}\left(\cot\theta +3\cot\theta \beta \right)}{\begin{array}{cc} & [\left(\frac{6}{5}+\frac{36}{5}\beta +\frac{45}{2}\beta^{2}+9\beta^{3}\right)+\frac{1}{F_{\rho }}\left(-\frac{2}{5}Mn-2Mn\beta -\frac{5}{2}Mn\beta^{2}-\frac{5}{2}\beta^{2}\right)\\ \; & +\frac{F_{\mu }}{F_{\rho }}\left(\frac{31}{30}MnPr+\frac{29}{8}\beta MnPr+4\beta^{2}MnPr+\frac{3}{2}\beta^{2}Mn\right)+\frac{F_{\mu }}{F_{\rho }^{2}}(-\frac{3}{8}Mn^{2}Pr\\ \; & -\frac{4}{3}\beta Mn^{2}Pr-\beta^{2}Mn^{2}Pr)] \end{array}}. \end{array}$$

When we assume that both the relative density and the relative viscosity of the nanofluid are 1, and the porous medium parameter and the Marangoni number are 0, that is, $F_{\rho }=F_{\mu }=1$ and β=0, Mn=0, the critical Reynolds number obtained at this time is consistent with the results of Benjamin and Yih.

## 4. Weakly Non-Linear Analysis

In order to predict the characteristics of thin film flow more precisely, we adopt a multiscale method and expand the interface perturbation $h_{1}$ in the following form:

(63) $$h_{1}\left(x,x_{1},\cdots ,t,t_{1},t_{2}\right)=\delta \eta_{1}+\delta^{2}\eta_{2}+\delta^{3}\eta_{3}+\cdots ,$$

where

(64) $$x_{1}=\delta x,t_{1}=\delta t,t_{2}=\delta^{2}t,\cdots ,$$

here *t* and *x* represent the rapidly varying scales, while $x_{1}$, $t_{1}$ represent the slowly varying scales. Assuming that these variables are independent of each other, the time and space derivatives become

(65) $$\begin{array}{cc} \; & \partial_{t}\to \partial_{t}+\delta \partial_{t_{1}}+\delta^{2}\partial_{t_{2}},\\ \; & \partial_{x}\to \partial_{x}+\delta \partial_{x_{1}}. \end{array}$$

Substituting Equations (63)–(65) into Equation (56), we can obtain

(66) $$\left(L_{0}+\delta L_{1}+\delta^{2}L_{2}+\cdots \right)\left(\delta \eta_{1}+\delta^{2}\eta_{2}+\delta^{3}\eta_{3}+\cdots \right)=-\delta^{2}N_{2}-\delta^{3}N_{3}-\cdots ,$$

where the expressions of the operators $L_{0}$, $L_{1}$, $L_{2}$ and the nonlinear terms $N_{2}$, $N_{3}$ in Equation (66) are as follows

$$\begin{array}{cc} L_{0}= & \frac{\partial }{\partial t}+A\frac{\partial }{\partial x}+B\frac{\partial^{2}}{\partial x^{2}}+D\frac{\partial^{4}}{\partial x^{4}},\\ L_{1}= & \frac{\partial }{\partial t_{1}}+A\frac{\partial }{\partial x_{1}}+2B\frac{\partial^{2}}{\partial x\partial x_{2}}+4D\frac{\partial^{4}}{\partial x^{3}\partial x_{1}},\\ L_{2}= & \frac{\partial }{\partial t_{2}}+B\frac{\partial^{2}}{\partial x_{1}^{2}}+6D\frac{\partial^{4}}{\partial x^{2}\partial x_{1}^{2}},\\ N_{2}= & A^{\prime }\eta_{1}\frac{\partial \eta_{1}}{\partial x}+B^{\prime }\left[\eta_{1}\frac{\partial^{2}\eta_{1}}{\partial x^{2}}+{\left(\frac{\partial \eta_{1}}{\partial x}\right)}^{2}\right]+C^{\prime }\left[\eta_{1}\frac{\partial^{4}\eta_{1}}{\partial x^{4}}+\frac{\partial \eta_{1}}{\partial x}\frac{\partial^{3}\eta_{1}}{\partial x^{3}}\right],\\ N_{3}= & A^{\prime }\left[\eta_{1}\left(\frac{\partial \eta_{2}}{\partial x}+\frac{\partial \eta_{1}}{\partial x_{1}}\right)+\eta_{2}\frac{\partial \eta_{1}}{\partial x}\right]+B^{\prime }[\eta_{1}\left(\frac{\partial^{2}\eta_{2}}{\partial x^{2}}+2\frac{\partial^{2}\eta_{1}}{\partial x\partial x_{1}}\right)+\eta_{2}\frac{\partial^{2}\eta_{1}}{\partial x^{2}}\\ \; & +\;2\frac{\partial \eta_{1}}{\partial x_{1}}\left(\frac{\partial \eta_{2}}{\partial x_{1}}+\frac{\partial \eta_{1}}{\partial x_{1}}\right)]+C^{\prime }[\eta_{1}\left(\frac{\partial^{4}\eta_{2}}{\partial x^{4}}+4\frac{\partial^{4}\eta_{1}}{\partial x^{3}\partial x_{1}}\right)+\eta_{2}\frac{\partial^{4}\eta_{1}}{\partial x^{4}}+\frac{\partial \eta_{1}}{\partial x}\\ \; & \times \;\left(\frac{\partial^{3}\eta_{2}}{\partial x^{3}}+3\frac{\partial^{3}\eta_{1}}{\partial x^{2}\partial x_{1}}\right)+\frac{\partial^{3}\eta_{1}}{\partial x^{3}}\left(\frac{\partial \eta_{2}}{\partial x}+\frac{\partial \eta_{1}}{\partial x_{1}}\right)]+\frac{1}{2}A^{\prime \prime }\eta_{1}^{2}\frac{\partial \eta_{1}}{\partial x}\\ \; & +\;B^{\prime \prime }\left[\frac{1}{2}\eta_{1}^{2}\frac{\partial^{2}\eta_{1}}{\partial x^{2}}+\eta_{1}{\left(\frac{\partial \eta_{1}}{\partial x}\right)}^{2}\right]+C^{\prime \prime }\left(\frac{1}{2}\eta_{1}^{2}\frac{\partial^{4}\eta_{1}}{\partial x^{4}}+\eta_{1}\frac{\partial \eta_{1}}{\partial x}\frac{\partial^{3}\eta_{1}}{\partial x^{3}}\right). \end{array}$$

For the first-order problem O(δ) of δ, we have:

(67) $$L_{0}\eta_{1}=0,$$

assume that the solution of this equation has the following form

(68) $$\eta_{1}=\Gamma \left(x_{1},t_{1},t_{2}\right)\left[\exp i\left(kx-\omega_{r}t\right)\right]+c.c.,$$

where $\Gamma (x_{1},\;t_{1},\;t_{2})$ represents the complex amplitude. It should be noted that in the linear stability analysis, we have already obtained the solution (67), except that ω is replaced by $\omega_{r}$, because near the neutral stability curve, the order of $\omega_{i}$ is O(δ). Therefore, the function $\exp(\omega_{i}t)$ changes slowly and can be absorbed by $\Gamma (x_{1},t_{1},t_{2})$.

For the second-order equation $O(\delta^{2})$

(69) $$L_{0}\eta_{2}=-L_{1}\eta_{1}-N_{2},$$

by substituting Equation (68) into Equation (69), we can obtain

(70) $$L_{0}\eta_{2}=-i\left[\frac{\partial D_{1}\left(\omega_{r},k\right)}{\partial \omega_{r}}\frac{\partial \Gamma }{\partial t_{1}}-\frac{\partial D_{1}\left(\omega_{r},k\right)}{\partial k}\frac{\partial \Gamma }{\partial x_{1}}\right]e^{i\left(kx-\omega_{r}t\right)}-\Omega \Gamma^{2}e^{2i\left(kx-\omega_{r}t\right)}+c.c.,$$

where

$$D_{1}\left(\omega_{r},k\right)=-i\omega_{r}+Aik-Bk^{2}+Ck^{4},$$

$$\Omega =iA^{\prime }k-2B^{\prime }k^{2}+2C^{\prime }k^{4}.$$

To ensure the validity and accuracy of the calculation results, we eliminate the secular term generated by the first term on the right side of Equation (70), thereby obtaining the expression of the solution $\eta_{2}$:

(71) $$\eta_{2}=-\frac{\Omega \Gamma^{2}e^{2i\left(kx-\omega_{r}t\right)}}{D_{1}\left(2\omega_{r},2k\right)}+c.c.,$$

by introducing the coordinate transformation $\xi =(x_{1}-c_{g}t_{1})$, where $c_{g}=\left(-D_{k}/D_{\omega r}\right)$, and utilizing the solvability condition of the third-order equation, the amplitude satisfies the following equation

(72) $$\frac{\partial \Gamma }{\partial t_{2}}+J_{1}\frac{\partial^{2}\Gamma }{\partial \xi^{2}}-\delta^{-2}\omega_{i}\Gamma +\left(J_{2}+iJ_{3}\right){\left|\Gamma \right|}^{2}\Gamma =0,$$

where the coefficients $J_{1}$, $J_{2}$, and $J_{3}$ are expressed as

$$J_{1}=B-6Ck^{2},$$

$$J_{2}=\frac{1}{2}\left(-B^{\prime \prime }k^{2}+C^{\prime \prime }k^{4}\right)+\left[\frac{{\left(A^{\prime }\right)}^{2}k^{2}-2\left(B^{\prime }k^{2}-7C^{\prime }k^{4}\right)\left(B^{\prime }k^{2}-C^{4}\right)}{16Ck^{4}-4Bk^{2}}\right],$$

$$J_{3}=\frac{1}{2}A^{\prime \prime }k+\left[\frac{A^{\prime }k\left(B^{\prime }k^{2}-7C^{\prime }k^{4}\right)+2A^{\prime }k\left(B^{\prime }k^{2}-C^{\prime }k^{4}\right)}{16Ck^{4}-4Bk^{2}}\right].$$

Ignoring the diffusion effect of the second term in Equation (72), we obtain

(73) $$\frac{\partial \Gamma }{\partial t_{2}}-\delta^{-2}\omega_{i}\Gamma +\left(J_{2}+iJ_{3}\right){\left|\Gamma \right|}^{2}\Gamma =0.$$

Assume that the solution of Equation (73) can be written in the following form

(74) $$\Gamma =a\exp\left[-ib\left(t_{2}\right)t_{2}\right].$$

Substituting Equation (74) into Equation (73), we get

(75) $$\frac{\partial a}{\partial t_{2}}=\left(\delta^{-2}\omega_{i}-J_{2}a^{2}\right)a,$$

(76) $$\frac{\partial \left[b\left(t_{2}\right)t_{2}\right]}{\partial t_{2}}=a^{2}J_{3}.$$

The second term on the right side of Equation (75) is a nonlinear term, and this term can adjust the exponential change of the linear perturbation according to the signs of $\omega_{i}$ and $J_{2}$. When $J_{2}=0$, Equation (75) simplifies to the equation given in the linear theory. When the right side of Equation (75) is zero, the equilibrium amplitude can be obtained as

(77) $$\delta a={\left(\frac{\omega_{i}}{J_{2}}\right)}^{1/2}.$$

When $J_{2}>0$, the amplitude reaches saturation; while when $J_{2}<0$, the amplitude does not saturate, that is, $J_{2}<0$ will lead to the instability of the system. According to the signs of $\omega_{i}$ and $J_{2}$, the following four nonlinear regions are defined: the supercritical stable region $S_{1}$ ($\omega_{i}>0,J_{2}>0$), the subcritical unstable region $S_{2}$ ($\omega_{i}<0,J_{2}<0$), the unconditionally stable region $S_{3}$ ($\omega_{i}<0,J_{2}>0$), and the explosive state region $S_{4}$ ($\omega_{i}>0,J_{2}<0$).

## 5. Numerical Simulations

Nonlinear evolution equations are vital mathematical tools for describing complex film dynamics. To solve the governing Equation (55) in a periodic domain, we employ a pseudospectral method based on the Fast Fourier Transform (FFT) algorithm. This approach transforms the spatial variables into a series of wavenumbers, converting the partial differential equation (PDE) into a coupled system of ordinary differential equations (ODEs).

Specifically, the film thickness h(x,t) is mapped to the spectral domain as $\hat{h}\left(k,t\right)=F\left\{h\left(x,t\right)\right\}$, where *F* and $F^{-1}$ denote the forward and inverse FFT operators, respectively. This technique allows spatial derivatives to be computed algebraically as $F\left\{\partial_{x}^{n}h\right\}={\left(ik\right)}^{n}\hat{h}$. To maintain computational efficiency and avoid expensive convolution sums, nonlinear terms are evaluated in the physical domain through point-wise multiplication after applying $F^{-1}$, before being transformed back to the spectral domain. Applying this procedure to Equation (55) yields the following evolution system:

(78) $$\begin{array}{cc} \frac{\partial \hat{h}}{\partial t}= & -ik\cdot F\{\frac{F_{\rho }}{F_{\mu }}F^{-1}{\left[\hat{h}\right]}^{3}+\left(\frac{3F_{\rho }}{F_{\mu }}\beta -\frac{Mn}{2F_{\mu }}\right)F^{-1}{\left[\hat{h}\right]}^{2}-\frac{Mn}{F_{\mu }}\beta F^{-1}\left[\hat{h}\right]+\frac{6F_{\rho }^{2}}{5F_{\mu }^{3}}ReF^{-1}{\left[\hat{h}\right]}^{6}\\ \; & \times \;F^{-1}\left[ik\hat{h}\right]+\left(\frac{36F_{\rho }^{2}}{5F_{\mu }^{3}}Re\beta -\frac{2F_{\rho }}{5F_{\mu }^{3}}MnRe+\frac{F_{\rho }}{30F_{\mu }^{2}}MnRePr\right)F^{-1}{\left[\hat{h}\right]}^{5}F^{-1}\left[ik\hat{h}\right]\\ \; & +\left(-\frac{2F_{\rho }}{F_{\mu }^{3}}MnRe\beta +\frac{45F_{\rho }^{2}}{2F_{\mu }^{3}}Re\beta^{2}+\frac{13F_{\rho }}{8F_{\mu }^{2}}\beta MnRePr-\frac{3Mn^{2}}{8F_{\mu }^{2}}RePr+\frac{F_{\rho }}{F_{\mu }^{2}}MnRePr\right)\\ \; & \times \;F^{-1}{\left[\hat{h}\right]}^{4}F^{-1}\left[ik\hat{h}\right]+(-\frac{5F_{\rho }}{2F_{\mu }^{3}}MnRe\beta^{2}+\frac{9F_{\rho }^{2}}{F_{\mu }^{3}}Re\beta^{3}-\frac{5F_{\rho }}{2F_{\mu }^{3}}Re\beta^{2}-\frac{F_{\rho }}{F_{\mu }}\cot\theta \\ \; & -\frac{4Mn^{2}}{3F_{\mu }^{2}}\beta RePr+\frac{4F_{\rho }}{F_{\mu }^{2}}\beta^{2}MnRePr+\frac{2F_{\rho }}{F_{\mu }^{2}}\beta MnRePr)F^{-1}{\left[\hat{h}\right]}^{3}F^{-1}\left[ik\hat{h}\right]\\ \; & +\left(\frac{3F_{\rho }}{2F_{\mu }^{2}}MnRe\beta^{2}-\frac{3F_{\rho }}{F_{\mu }}\cot\theta \beta -\frac{Mn^{2}}{F_{\mu }^{2}}\beta^{2}RePr\right)F^{-1}{\left[\hat{h}\right]}^{2}F^{-1}\left[ik\hat{h}\right]\\ \; & +\frac{1}{3Ca^{\prime }F_{\mu }}F^{-1}{\left[\hat{h}\right]}^{3}F^{-1}\left[{\left(ik\right)}^{3}\hat{h}\right]+\frac{\beta }{Ca^{\prime }F_{\mu }}F^{-1}{\left[\hat{h}\right]}^{2}F^{-1}\left[{\left(ik\right)}^{3}\hat{h}\right]\}. \end{array}$$

For the numerical setup, the computational domain is set to L=14π/0.28, ensuring that multiple wavelengths of the most unstable mode are captured. The domain is discretized with N=100 grid points, a resolution verified through grid-independence tests to ensure that truncation errors remain negligible. The initial condition is prescribed as a small harmonic perturbation $h\left(x,0\right)=1+0.05\cos\left(k_{m}x\right)$, where $k_{m}$ is the most unstable wavenumber determined by linear stability analysis.

Temporal integration of Equation (78) is performed using the MATLAB ode45 solver, an adaptive-step scheme based on the explicit fourth- and fifth-order Runge-Kutta method. Unlike fixed-step methods, ode45 automatically adjusts the internal integration step to satisfy a relative error tolerance of $10^{-6}$, ensuring numerical stability during rapid nonlinear deformation. The time increment for data output is fixed at 0.1, which is sufficient to resolve the intricate interfacial features of the film.

## 6. Results and Discussion

### 6.1. Linear Stability Analysis

**Figure 2:**
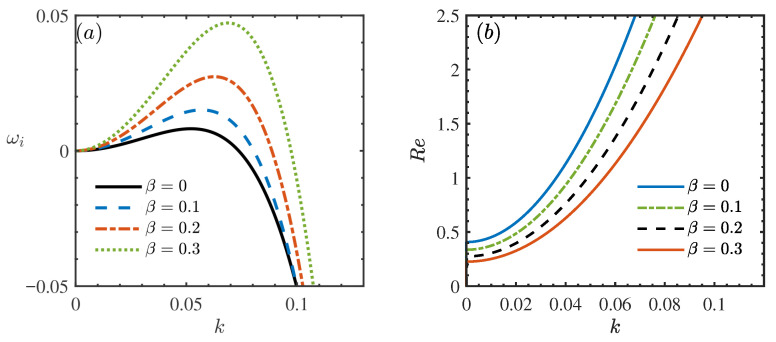
(**a**) Dispersion relation for the long-wave model when $\theta =45^{\circ }$, Re=2, ϕ=0.05, $\zeta_{0}=2$, Mn=0.4, Pr=7. (**b**) Neutral stability curves for different β when $\theta =45^{\circ }$, ϕ=0.05, $\zeta_{0}=2$, Mn=0.2, Pr=7.

Figure 2 illustrates the impact of the porous medium’s permeability parameter, β, on the stability of the nanofluid flow, presenting both the dispersion relation (Figure 2a) and the neutral stability curves (Figure 2b). In Figure 2a, the growth rate of linear perturbations, $\omega_{i}$, is plotted against the wave number *k*. The flow is considered stable when $\omega_{i}<0$, unstable when $\omega_{i}>0$, and neutrally stable at $\omega_{i}=0$. As β increases, the dispersion curves shift upward, signifying an enhancement of flow instability. Physically, a higher β corresponds to increased permeability of the porous substrate, which reduces flow resistance and weakens the stabilizing interaction between the fluid and the wall, thereby making the flow more susceptible to disturbances. This destabilizing effect is further corroborated in Figure 2b, where neutral stability curves are plotted in the (k,Re) plane. Here, the region below each curve represents unstable flow conditions. The intersection of each curve with the vertical axis (k=0) defines the critical Reynolds number ($Re_{c}$). The results indicate that an increase in β leads to a decrease in $Re_{c}$, confirming that higher permeability destabilizes the film flow. This finding aligns with the conclusions reported by Sadiq and Usha [36], reinforcing the validity of the current model. In practical applications such as liquid film cooling, excessive permeability could therefore intensify fluctuations, potentially compromising system performance.

**Figure 3:**
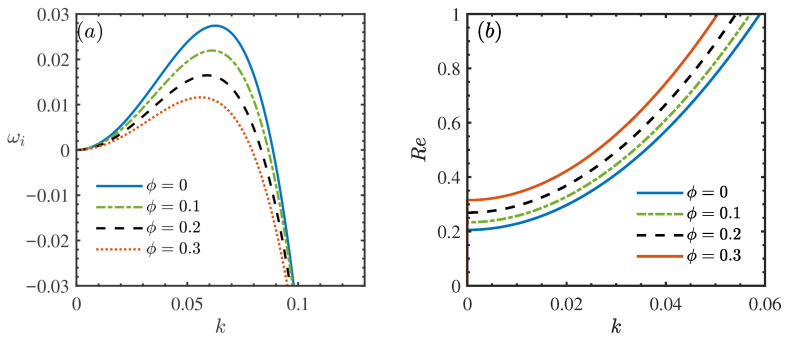
(**a**) Dispersion relation for the long-wave model when $\theta =45^{\circ }$, Re=2, β=0.2, $\zeta_{0}=2$, Mn=0.4, Pr=7. (**b**) Neutral stability curves for different ϕ when $\theta =45^{\circ }$, β=0.2, $\zeta_{0}=2$, Mn=0.2, Pr=7.

Figure 3 delineates the influence of the nanoparticle volume fraction, ϕ, on the hydrodynamic stability of the film flow. Figure 3a reveals that the peak growth rate $\omega_{i}$ decreases monotonically as ϕ increases, indicating a strong stabilizing effect. This trend is corroborated by the neutral stability curves in Figure 3b, where the critical Reynolds number ($Re_{c}$) shifts markedly towards higher values with increasing ϕ. Physically, this stabilization stems from the modified transport properties of the nanofluid. The addition of nanoparticles elevates the effective viscosity, intensifying the dissipation of kinetic energy within disturbance waves [33,37]. Consequently, the nanofluid system requires significantly larger inertial forces to trigger instability compared to the base fluid.

**Figure 4:**
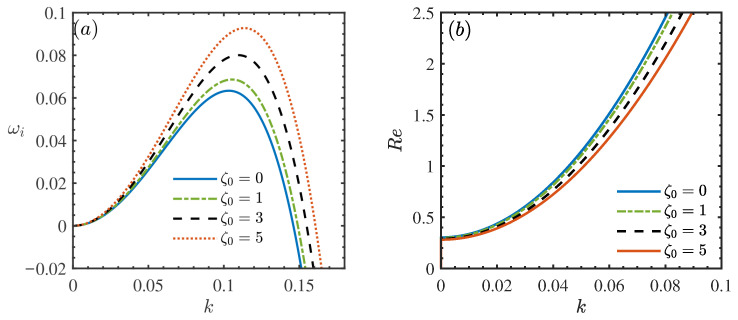
(**a**) Dispersion relation for the long-wave model when $\theta =45^{\circ }$, Re=2, β=0.2, ϕ=0.05, Mn=0.4, Pr=7. (**b**) Neutral stability curves for different $\zeta_{0}$ when $\theta =45^{\circ }$, β=0.2, ϕ=0.05, Mn=0.2, Pr=7.

Figure 4 elucidates the impact of the density ratio parameter, $\zeta_{0}$, on the hydrodynamic stability of the nanofluid film. Figure 4a presents the dispersion relation, revealing that the peak growth rate $\omega_{i}$ increases monotonically as $\zeta_{0}$ rises from 0 to 5. This trend indicates that a larger density difference between the nanoparticles and the base fluid exerts a destabilizing effect on the flow. Physically, this phenomenon stems from the alteration of the gravitational body force acting on the fluid. The parameter $\zeta_{0}$ characterizes the relative density contribution of the nanoparticles; a higher $\zeta_{0}$ implies a denser nanofluid mixture. Since the film flow is gravity-driven, an increased effective density amplifies the streamwise component of gravity [7]. This enhanced driving force augments the fluid’s momentum and the associated inertial forces. According to classical stability theory, inertia promotes the amplification of surface waves, while viscosity acts as a stabilizing damping force [8]. Consequently, when the density-induced inertial effects overwhelm viscous damping, the flow becomes more susceptible to disturbances. This conclusion is quantitatively corroborated by the neutral stability curves in Figure 4b, where the critical Reynolds number ($Re_{c}$) decreases significantly as $\zeta_{0}$ increases. This reduction in $Re_{c}$ confirms that the presence of heavier nanoparticles lowers the threshold for the onset of instability, thereby expanding the unstable domain.

**Figure 5:**
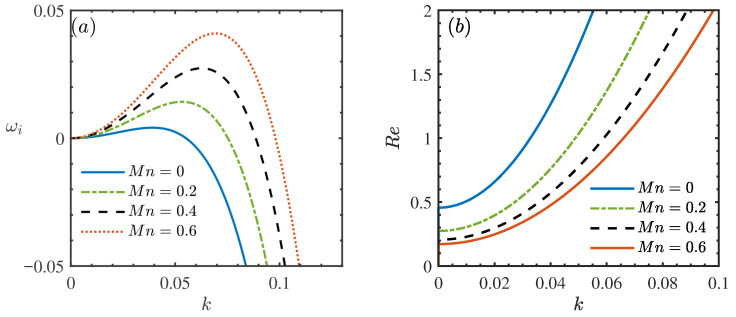
(**a**) Dispersion relation for the long-wave model when $\theta =45^{\circ }$, Re=2, β=0.2, ϕ=0.05, $\zeta_{0}=2$, Pr=7. (**b**) Neutral stability curves for different Mn when $\theta =45^{\circ }$, β=0.2, ϕ=0.05, $\zeta_{0}=2$, Pr=7.

Figure 5 illustrates the influence of the Marangoni number, Mn, on the hydrodynamic stability of the film flow. Figure 5a presents the dispersion relation between the growth rate $\omega_{i}$ and the wavenumber *k*. It is observed that as Mn increases, the dispersion curve shifts noticeably upward, resulting in a higher peak growth rate. This trend indicates that the Marangoni effect acts as a destabilizing factor in the flow system. This conclusion is further corroborated by the neutral stability curves in Figure 5b, where an increase in Mn leads to a significant reduction in the stable region and a decrease in the critical Reynolds number ($Re_{c}$). Physically, this destabilization is driven by the thermocapillary mechanism [38]. In a heated falling film, a local thinning of the liquid layer (wave trough) results in a higher surface temperature due to its proximity to the heated wall, while thicker regions (wave crests) remain relatively cooler. Since surface tension decreases with increasing temperature, a surface tension gradient is established along the interface. This gradient generates a thermocapillary stress that drags the fluid from the hotter, low-surface-tension troughs toward the cooler, high-surface-tension crests [39]. This mass transport exacerbates the surface deformation, thereby amplifying the disturbances and promoting flow instability [36].

### 6.2. Weakly Non-Linear Stability Analysis

**Figure 6:**
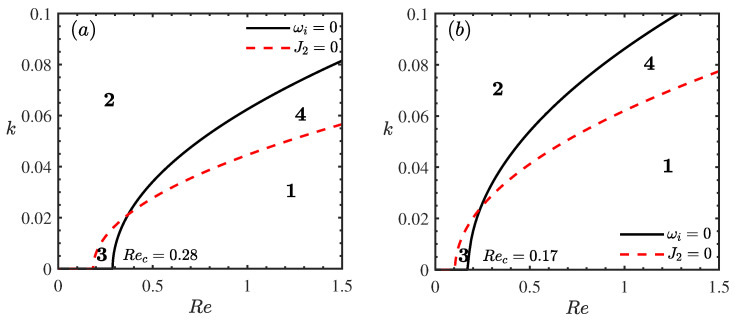
Stability curves *k* vs. Re for different β when $\theta =45^{\circ }$, Mn=0.4, ϕ=0.05, $\zeta_{0}=5$, Pr=7. (**a**) β=0, (**b**) β=3. The regions labeled 1, 2, 3, and 4 represent the supercritical stable ($S_{1}$), subcritical unstable ($S_{2}$), unconditionally stable ($S_{3}$), and explosive instability ($S_{4}$) regimes, respectively.

Figure 6 delineates the stability boundaries in the (Re,k) plane for varying porous medium permeabilities β. The domain is partitioned into four distinct regimes by the neutral stability curve ($\omega_{i}=0$) and the threshold curve where the Landau coefficient vanishes ($J_{2}=0$). These regimes are identified as follows: the supercritical stable region $S_{1}$ ($\omega_{i}>0,J_{2}>0$), where finite-amplitude waves saturate to a stable equilibrium; the subcritical unstable region $S_{2}$ ($\omega_{i}<0,J_{2}<0$), where the flow is linearly stable but unstable to finite-amplitude disturbances; the unconditionally stable region $S_{3}$ ($\omega_{i}<0,J_{2}>0$); and the explosive instability region $S_{4}$ ($\omega_{i}>0,J_{2}<0$). Comparing Figure 6a,b, it is evident that an increase in permeability β leads to a reduction in the critical Reynolds number ($Re_{c}$). Crucially, increasing β significantly expands the explosive region $S_{4}$ while shrinking the supercritical stable region $S_{1}$. Physically, this implies that higher permeability not only lowers the threshold for linear instability but also promotes a more dangerous type of instability where disturbance amplitudes may grow unbounded, potentially accelerating the transition to turbulence. This confirms that the permeability of the inclined plane has a profound destabilizing effect on the flow field in both linear and nonlinear regimes.

**Figure 7:**
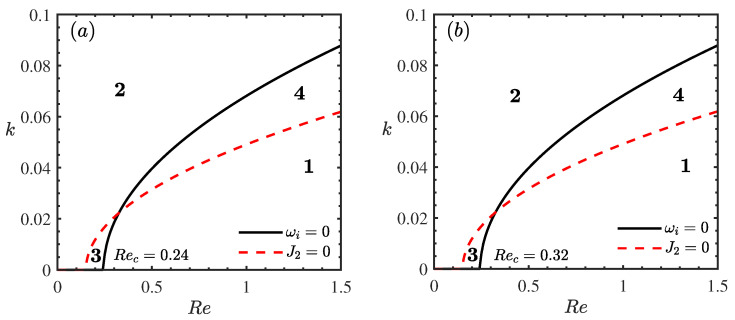
Stability curves *k* vs. Re for different ϕ when $\theta =45^{\circ }$, Mn=0.4, β=0.1, $\zeta_{0}=5$, Pr=7. (**a**) ϕ=0, (**b**) ϕ=0.5. The regions labeled 1, 2, 3, and 4 represent the supercritical stable ($S_{1}$), subcritical unstable ($S_{2}$), unconditionally stable ($S_{3}$), and explosive instability ($S_{4}$) regimes, respectively.

Figure 7 presents the weakly nonlinear stability diagrams in the (Re,k) plane for different nanoparticle volume fractions ϕ. A comparative analysis of Figure 7a (ϕ=0) and Figure 7b (ϕ=0.5) reveals a marked stabilizing trend: the critical Reynolds number ($Re_{c}$) increases from approximately 0.24 to 0.32. This shift significantly expands the unconditionally stable region ($S_{3}$). Crucially, from a nonlinear perspective, the explosive instability region ($S_{4}$), where disturbances are predicted to grow unboundedly potentially leading to film rupture, is notably compressed. Physically, this suppression of nonlinear instability is governed by the enhanced energy dissipation mechanism inherent to nanofluids. In the weakly nonlinear regime, the evolution of the disturbance amplitude is dictated by the balance between energy supply and energy dissipation. The addition of nanoparticles elevates the effective viscosity of the fluid [32]. This enhancement intensifies the viscous damping of finite-amplitude waves, effectively counteracting the nonlinear energy transfer that typically drives the system towards explosive growth. Consequently, the threshold for subcritical instability is raised, and the nanofluid film is more likely to saturate into stable finite-amplitude traveling waves rather than undergoing catastrophic rupture, exhibiting superior global stability compared to the pure base fluid [40].

**Figure 8:**
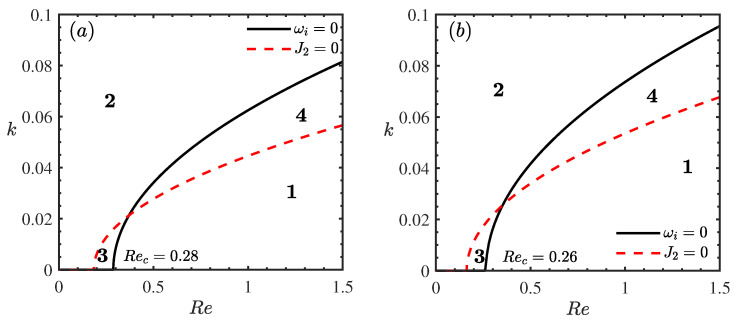
Stability curves *k* vs. Re for different $\zeta_{0}$ when $\theta =45^{\circ }$, Mn=0.4, β=0.1, ϕ=0.2, Pr=7. (**a**) $\zeta_{0}=0$, (**b**) $\zeta_{0}=5$. The regions labeled 1, 2, 3, and 4 represent the supercritical stable ($S_{1}$), subcritical unstable ($S_{2}$), unconditionally stable ($S_{3}$), and explosive instability ($S_{4}$) regimes, respectively.

Figure 8 presents the weakly nonlinear stability diagrams in the (Re,k) plane for varying density ratio parameters $\zeta_{0}$. A comparison between Figure 8a ($\zeta_{0}=0$) and Figure 8b ($\zeta_{0}=5$) reveals that the critical Reynolds number ($Re_{c}$) decreases noticeably from 0.30 to 0.26. Concurrently, the topology of the stability map undergoes a significant transformation: the unconditionally stable region ($S_{3}$) shrinks, whereas the explosive instability region ($S_{4}$) expands substantially. Physically, this global destabilization is driven by the enhanced inertial effects associated with heavier nanoparticles. An increase in $\zeta_{0}$ signifies a larger density difference between the particles and the base fluid, which amplifies the effective gravitational component driving the flow [7]. This increased driving force results in higher local momentum and stronger inertial forces. In the nonlinear regime, these inertial forces facilitate the energy transfer from the mean flow to the disturbance waves. When this energy input overwhelms the viscous dissipation, finite-amplitude disturbances grow unboundedly. The expansion of the explosive region ($S_{4}$) specifically indicates that the nanofluid film becomes increasingly susceptible to catastrophic rupture even at lower Reynolds numbers, as the stabilizing viscous forces are outmatched by the density-induced inertia [32].

**Figure 9:**
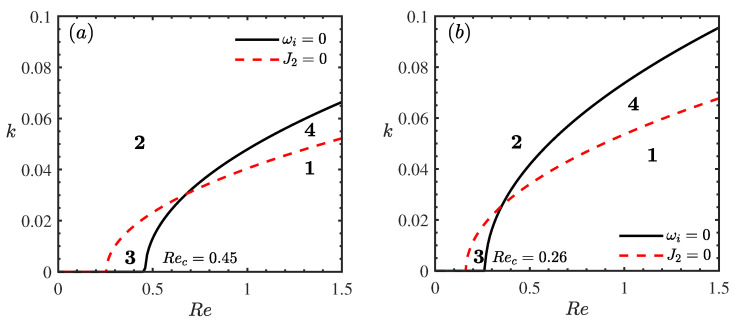
Stability curves *k* vs. Re for different Mn when $\theta =45^{\circ }$, $\zeta_{0}=5$, β=0.1, ϕ=0.2, Pr=7. (**a**) Mn=0, (**b**) Mn=0.4. The regions labeled 1, 2, 3, and 4 represent the supercritical stable ($S_{1}$), subcritical unstable ($S_{2}$), unconditionally stable ($S_{3}$), and explosive instability ($S_{4}$) regimes, respectively.

Figure 9 delineates the weakly nonlinear stability diagrams in the (Re,k) plane for varying Marangoni numbers Mn. A comparative assessment reveals that as Mn increases, the critical Reynolds number ($Re_{c}$) decreases significantly. Concurrently, the topology of the stability map transforms drastically: the unconditionally stable region ($S_{3}$) shrinks, while the explosive instability region ($S_{4}$) expands substantially. Physically, this profound destabilization is driven by the intensification of thermocapillary stresses at the free surface. The Marangoni effect induces a flow from the hotter, thinner regions (wave troughs) to the cooler, thicker regions (wave crests) due to surface tension gradients [38]. In the weakly nonlinear regime, this mass transport reinforces surface deformations. When Mn is high, the energy input from thermocapillary forces overwhelms the viscous dissipation that typically saturates the wave amplitude. The significant expansion of the explosive region ($S_{4}$) indicates that strong Marangoni effects create a self-amplifying feedback loop, rendering the nanofluid film highly susceptible to unbounded disturbance growth and potential rupture, even at relatively low Reynolds numbers [32].

### 6.3. Numerical Simulations

**Figure 10:**
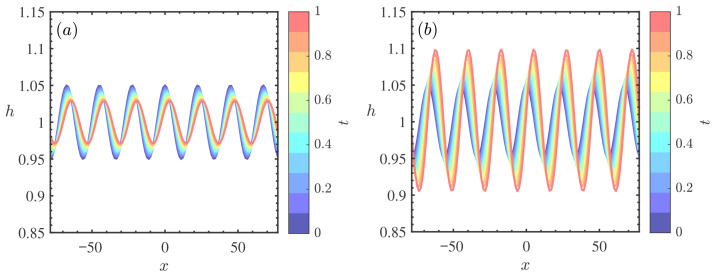
Film thickness at different times when $\theta =45^{\circ }$, $\zeta_{0}=5$, Re=10, k=0.28, ϕ=0.2, Mn=0.4, Pr=7. (**a**) β=0, (**b**) β=0.3.

Figure 10 illustrates the spatiotemporal evolution of the interfacial waves obtained from the direct numerical simulation for Re=10 within the time span t∈[0,1]. The color gradient represents the progression of time, revealing that the amplitude of the surface elevation h(x,t) grows significantly as time evolves. This confirms that at Re=10, the flow is in the unstable regime, where small perturbations evolve into finite-amplitude traveling waves. Comparing the wave profiles in Figure 10a (β=0) and Figure 10b (β=0.3), a distinct difference in the nonlinear growth is observed. For the solid substrate (β=0), the wave amplitude reaches a maximum of approximately 1.05. In contrast, for the porous substrate with β=0.3, the wave crests are noticeably amplified, reaching a dimensionless height of roughly 1.10. This numerical evidence aligns perfectly with the predictions from the linear and weakly nonlinear stability analyses presented earlier. It demonstrates that an increase in permeability β reduces the wall friction and enhances the momentum transfer near the interface, thereby accelerating the growth of disturbances and intensifying the flow instability in the nonlinear regime. This finding is consistent with the results of Sadiq et al. [41], who reported that the presence of a porous substrate and higher permeability significantly increases the amplitude of disturbance waves in heated liquid films.

**Figure 11:**
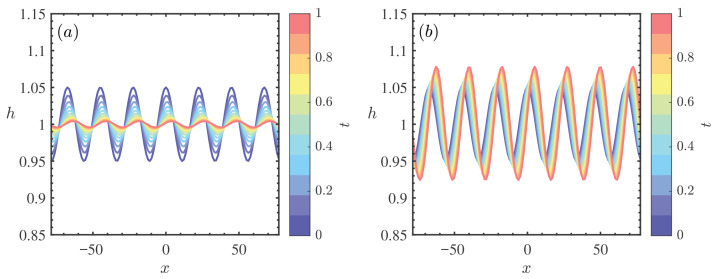
Film thickness at different times when $\theta =45^{\circ }$, $\zeta_{0}=5$, Re=10, k=0.28, β=0.25, Mn=0.4, Pr=7. (**a**), ϕ=0; (**b**), ϕ=0.3.

Figure 11 depicts the spatiotemporal evolution of the interfacial waves for different nanoparticle volume fractions ϕ at Re=10. Similar to the observations in Figure 10, the temporal color gradient confirms that the flow is in the unstable nonlinear regime, characterized by the growth of surface disturbances into finite-amplitude waves. A comparative analysis of Figure 11a (ϕ=0) and Figure 11b (ϕ=0.3) reveals a notable impact of the nanoparticle concentration on the wave dynamics. It is observed that the wave amplitude in the nanofluid (ϕ=0.3) is significantly larger than that in the pure fluid (ϕ=0). Specifically, the wave crests in Figure 11b reach higher dimensionless values, indicating a more intense nonlinear interaction. Physically, this phenomenon can be attributed to the competition between viscous damping and inertial forces. Although increasing ϕ enhances the effective viscosity (a stabilizing factor), the presence of heavy nanoparticles (indicated by a high density ratio $\zeta_{0}=5$) concurrently increases the effective density of the mixture. At the relatively high Reynolds number of Re=10, the density-induced inertial destabilization dominates over the viscous stabilization. Consequently, a higher concentration of heavy nanoparticles fuels the momentum of the disturbance waves, leading to larger amplitudes and a more unstable interface [37,41].

**Figure 12:**
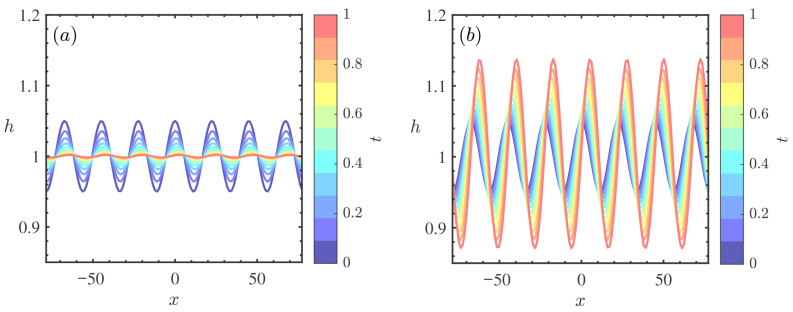
Film thickness at different times when $\theta =45^{\circ }$, β=0.3, Re=10, k=0.28, ϕ=0.3, Mn=0.4, Pr=7. (**a**) $\zeta_{0}=0$, (**b**) $\zeta_{0}=5$.

Figure 12 illustrates the spatiotemporal evolution of the interfacial waves for different density ratio parameters $\zeta_{0}$ at Re=10. Consistent with the previous cases, the temporal color gradient indicates that the flow is in the unstable nonlinear regime, where disturbances evolve into sustained finite-amplitude traveling waves. A comparison between Figure 12a ($\zeta_{0}=0$) and Figure 12b ($\zeta_{0}=5$) reveals a positive correlation between the transient wave amplitude and the density ratio. Specifically, the wave crests for $\zeta_{0}=5$ exhibit significantly larger dimensionless amplitudes compared to the case with $\zeta_{0}=0$. Physically, this amplification is governed by the density-induced inertial mechanism. The parameter $\zeta_{0}$ characterizes the relative density of the nanoparticles; a higher $\zeta_{0}$ corresponds to a heavier nanofluid mixture. Since the flow is gravity-driven, an increase in effective density amplifies the streamwise component of the gravitational body force [7]. This enhanced driving force imparts greater momentum to the fluid, thereby intensifying the inertial forces within the wave crests. In the nonlinear regime, this elevated inertia effectively overcomes the viscous damping, fueling the energy transfer from the mean flow to the disturbance and resulting in larger wave amplitudes [32]. This confirms that the density difference exerts a profound destabilizing influence on the film flow evolution.

**Figure 13:**
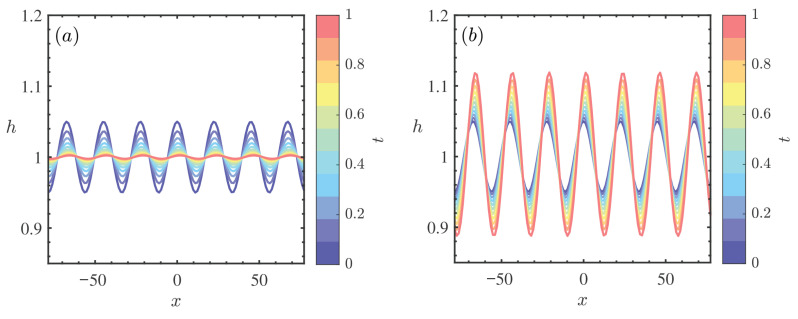
Film thickness at different times when $\theta =45^{\circ }$, $\zeta_{0}=0.5$, Re=35, k=0.28, ϕ=0.5, β=0.4, Pr=7. (**a**) Mn=0, (**b**) Mn=0.8.

Figure 13 depicts the spatiotemporal evolution of the interfacial waves for different Marangoni numbers Mn at a Reynolds number of Re=35. The simulation results clearly indicate that the flow is in the highly nonlinear unstable regime, where infinitesimal disturbances have evolved into large-amplitude traveling waves. A comparison between Figure 13a (Mn=0) and Figure 13b (Mn=0.8) reveals the profound destabilizing impact of the Marangoni effect. In the absence of thermocapillary forces (Mn=0), the wave maintains a moderate amplitude. However, when Mn is increased to 0.8, the wave profiles undergo a dramatic amplification, characterized by sharper crests and significantly larger peak-to-trough heights. Physically, this intensification is driven by the thermocapillary stress induced by surface temperature gradients. In a heated film, the wave troughs are closer to the heated wall and thus hotter than the crests. Consequently, surface tension is lower in the troughs and higher at the crests. This gradient generates a tangential stress that pulls liquid from the troughs toward the crests, reinforcing the surface deformation [38]. At high Reynolds numbers (Re=35), this mechanism acts in concert with inertial forces to overcome viscous damping, leading to the severe amplification of disturbance waves observed in the simulation [39,41].

## 7. Conclusions

This study provides a comprehensive investigation into the stability of nanofluid thin film flow over a non-uniformly heated, inclined porous substrate subject to a slip boundary condition. A nonlinear evolution equation governing the film thickness was derived utilizing the long-wave approximation and the Beavers-Joseph slip model. By systematically employing linear stability analysis, weakly nonlinear analysis, and direct numerical simulations via the Fast Fourier Transform (FFT) method, we dissected the specific influences of porous medium permeability, nanoparticle volume fraction, density ratio, and the Marangoni number on flow dynamics.

The results from the linear stability analysis reveal distinct competitive mechanisms governing film stability. The porous medium permeability (β), nanoparticle-to-base-fluid density ratio ($\zeta_{0}$), and Marangoni number (Mn) were identified as destabilizing factors. Specifically, increasing the porous parameter β from 0 to 0.3 and the Marangoni number Mn from 0 to 0.3 significantly amplifies the maximum temporal growth rate of perturbations and expands the unstable wavenumber domain, effectively reducing the critical Reynolds number ($Re_{c}$). Similarly, a higher density ratio ($\zeta_{0}$ increasing from 0 to 5) enhances the instability growth rate. In stark contrast, the nanoparticle volume fraction (ϕ) acts as a dominant stabilizing agent; increasing ϕ from 0 to 0.3 markedly suppresses the growth rate and shifts the neutral stability curve towards higher Reynolds numbers due to increased effective viscosity. Furthermore, both the weakly nonlinear analysis and the FFT-based numerical simulations show excellent agreement with these linear predictions.

These findings offer critical theoretical guidance for engineering applications dependent on stable thin films, such as micro-scale liquid cooling and precision coating techniques (e.g., CVD or PVD processes). To enhance film uniformity, our model suggests optimizing the nanoparticle concentration and minimizing the density mismatch between particles and the base fluid. Conversely, destabilizing effects can be mitigated by controlling the substrate permeability and managing thermal gradients to limit thermocapillary forces. In summary, this work demonstrates that the stability of nanofluid films on complex substrates can be precisely tailored by balancing these competing physical mechanisms. Future research could extend this framework to consider particle aggregation kinetics, non-Newtonian rheological behaviors, and three-dimensional instability patterns.

## Data Availability

The data that support the findings of this study are available from the corresponding author upon reasonable request.

## Appendix A

Equations (9) and (10) originate from the stress balance conditions imposed at the deformable free surface, which is described by

(A1) y=h(x,t),

The corresponding unit normal and tangential vectors are given by

(A2) $$n=\frac{\left(-h_{x},1\right)}{\sqrt{1+h_{x}^{2}}},\;t=\frac{\left(1,h_{x}\right)}{\sqrt{1+h_{x}^{2}}},$$

where $h_{x}=\partial h/\partial x$ denotes the local slope of the interface.

For a Newtonian nanofluid, the stress tensor in the liquid phase reads

(A3) $$\tau^{\left(l\right)}=-pI+\mu_{n}\left(\nabla u+\nabla u^{T}\right),$$

with u=(u,v) being the velocity field, *p* the pressure, and $\mu_{n}$ the dynamic viscosity. In two dimensions, the stress components are

(A4) $$\begin{array}{cc} \tau_{xx} & =-p+2\mu_{n}\frac{\partial u}{\partial x},\\ \tau_{yy} & =-p+2\mu_{n}\frac{\partial v}{\partial y},\\ \tau_{xy} & =\mu_{n}\left(\frac{\partial u}{\partial y}+\frac{\partial v}{\partial x}\right). \end{array}$$

At the free surface, the gas-phase viscous stresses are neglected, and only the gas pressure $p_{g}$ is retained. The tangential stress balance can thus be written as

(A5) $$t\cdot \left(\tau^{\left(l\right)}-\tau^{\left(g\right)}\right)\cdot n=\nabla_{s}\gamma ,$$

where $\tau^{\left(l\right)}$ and $\tau^{\left(g\right)}$ denote the stress tensors in the liquid and gas phases, respectively, and $\nabla_{s}\gamma$ denotes the surface gradient of the surface tension.

Substituting the stress components together with the expressions for t and n yields

(A6) $$\frac{1}{\sqrt{1+h_{x}^{2}}}\left[\mu_{n}\left(1-h_{x}^{2}\right)\left(\frac{\partial v}{\partial x}+\frac{\partial u}{\partial y}\right)+2\mu_{n}h_{x}\left(\frac{\partial v}{\partial y}-\frac{\partial u}{\partial x}\right)\right]=\frac{\partial \gamma }{\partial x}+h_{x}\frac{\partial \gamma }{\partial y},$$

which corresponds to Equation (9).

Similarly, the normal stress balance at the interface is expressed as

(A7) $$n\cdot (\tau^{\left(l\right)}-\tau^{\left(g\right)})\cdot n=\gamma \kappa ,$$

where κ is the interface curvature. For a two-dimensional free surface,

(A8) $$\kappa =\frac{h_{xx}}{{\left(1+h_{x}^{2}\right)}^{3/2}}.$$

After projecting the stress tensor onto the normal direction, one obtains

(A9) $$p_{g}-p+\frac{\mu_{n}}{1+h_{x}^{2}}\left[2\left(1-h_{x}^{2}\right)\frac{\partial v}{\partial y}-2h_{x}\left(\frac{\partial u}{\partial y}+\frac{\partial v}{\partial x}\right)\right]=\gamma \frac{h_{xx}}{{\left(1+h_{x}^{2}\right)}^{3/2}},$$

which is Equation (10).

## References

[1] Chattopadhyay S., Subedar G.Y., Gaonkar A.K., Barua A.K., Mukhopadhyay A. (2022). Effect of odd-viscosity on the dynamics and stability of a thin liquid film flowing down on a vertical moving plate. Int. J. Nonlinear Mech..

[2] Craster R.V., Matar O.K. (2009). Dynamics and stability of thin liquid films. Rev. Mod. Phys..

[3] Kalliadasis S., Kiyashko A., Demekhin E.A. (2003). Marangoni instability of a thin liquid film heated from below by a local heat source. J. Fluid Mech..

[4] Chen C.I., Chen C.K., Yang Y.T. (2003). Weakly nonlinear stability analysis of thin viscoelastic film flowing down in the outer surface of a rotating vertical cylinder. Int. J. Eng. Sci..

[5] Sarka S., Ganguly S., Dutta P. (2017). Magnetohydrodynamic stationary and oscillatory convective stability in a mushy layer during binary alloy solidification. Appl. Math. Model..

[6] Sarkar S., Ganguly S., Mishra M. (2019). Single diffusive magnetohydrodynamic pressure driven miscible displacement flows in a channel. Phys. Fluids.

[7] Benjamin T.B. (1957). Wave formation in laminar flow down an inclined plane. J. Fluid Mech..

[8] Yih C.-S. (1963). Stability of liquid flow down an inclined plane. Phys. Fluids.

[9] Beavers G.S., Joseph D.D. (1967). Boundary conditions at a naturally permeable wall. J. Fluid Mech..

[10] Pascal J.P. (1999). Linear stability of fluid flow down a porous inclined plane. J. Phys. D Appl. Phys..

[11] Whitaker S. (1986). Flow in porous media—I: A theoretical derivation of Darcy’s law. Transp. Porous Med..

[12] Mohamad A.M., Yadav D., Awasthi M.K., Ragoju R., Hassan M. (2025). Heat and mass transfers on the chemically reactive thermosolutal convective flow of Rivlin-Ericksen fluid over a porous medium with viscous dissipation effect. J. Comput. Appl. Mech..

[13] Yadav D., Awasthi M.K., Ragoju R., Bhattacharyya K., Hassan M., Singh A.K., Wang J. (2025). Analytical and numerical simulation of temperature reliant thermal conductivity and viscosity disparities effects on the onset of cellular convective motion in a viscoelastic Oldroyd-B type fluid saturated permeable layer. J. Porous Media.

[14] Yadav D., Awasthi M.K., Mohamad A.M., Ragoju R., Bhattacharyya K., Hassan M. (2024). The onset of Casson fluid convection in a permeable medium layer produced by purely inner heating with magnetic field. J. Comput. Appl. Mech..

[15] Liu Q.S., Liu B. (2019). Stability of a liquid film flowing down a porous inclined plane. Phys. Rev. E.

[16] Hill A.A., Straughan B. (2008). Poiseuille flow instability in a fluid overlaying a porous medium. J. Fluid Mech..

[17] Lin J.Z., Xia Y., Ku X.K. (2016). Flow and heat transfer characteristics of nanofluids containing rod-like particles in a turbulent pipe flow. Int. J. Heat Mass Transf..

[18] Xiong X.P., Chen S., Yang B. (2017). Natural convection of SiO_2_-water nanofluid in square cavity with thermal square column. Appl. Math. Mech.-Engl. Ed..

[19] Abbasov T. (2014). Specific features of the thermal conductivity of nanofluids. J. Dispers. Sci. Technol..

[20] Huo Y., Zhang L. (2015). Experimental investigation of the dynamic viscosity of Al_2_O_3_-MEPCM nanofluids. Int. Commun. Heat Mass Transf..

[21] Esfe M.H., Saedodin S., Wongwises S., Toghraie D. (2015). Experimental investigation and modeling of dynamic viscosity of Ag-MgO/water hybrid nanofluid at various temperatures and solid volume fractions. Int. Commun. Heat Mass Transf..

[22] Lin J.Z., Xia Y., Bao F.B. (2014). Hydrodynamic instability of nanofluids in a channel flow. Fluid Dyn. Res..

[23] Zhang Y., Zhang M., Bai Y. (2017). Unsteady flow and heat transfer of power-law nanofluid thin film over a stretching sheet with variable magnetic field and power-law velocity slip effect. J. Taiwan Inst. Chem. Eng..

[24] Chand R., Yadav D., Bhattacharyya K., Awasthi M.K. (2021). Thermal convection in a layer of micropolar nanofluid. Asia-Pac. J. Chem. Eng..

[25] Lin S.P. (1975). Stability of liquid flow down a heated inclined plane. Lett. Heat Mass Transf..

[26] Samanta A. (2008). Stability of liquid film falling down a vertical non-uniformly heated wall. Phys. D.

[27] Samanta A. (2008). Stability of inertialess liquid film flowing down a heated inclined plane. Phys. Lett. A.

[28] Kirkinis E., Andreev A.V. (2019). Odd-viscosity-induced stabilization of viscous thin liquid films. J. Fluid Mech..

[29] Mukhopadhyay S., Mukhopadhyay A. (2020). Hydrodynamics and instabilities of falling liquid film over a non-uniformly heated inclined wavy bottom. Phys. Fluids.

[30] Mukhopadhyay A., Haldar S. (2010). Long-Wave Instabilities of Viscoelastic Fluid Film Flowing Down an Inclined Plane with Linear Temperature Variation. Z. Naturforsch. A.

[31] Benney D.J. (1966). Long waves on liquid films. J. Math. Phys..

[32] Oron A., Davis S.H., Bankoff S.G. (1997). Long-scale evolution of thin liquid films. Rev. Mod. Phys..

[33] Brinkman H.C. (1952). The viscosity of concentrated suspensions in solutions. J. Chem. Phys..

[34] Einstein A. (1906). Eine neue bestimung der molekuldimensionen. Ann. Phys..

[35] Pak B.C., Cho Y.I. (1998). Hydrodynamic and heat transfer study of dispersed fluids with submicron metallic oxide particles. Exp. Heat Transfer.

[36] Sadiq M.R., Usha R. (2008). Thin Newtonian film flow down a porous inclined plane: Stability analysis. Phys. Fluids.

[37] Maleki H., Nazari M., Kayhani M.H., Ahmadi G. (2020). Linear stability analysis of a nanofluid film flowing down an inclined plane with variable viscosity. Phys. Fluids.

[38] Pearson J.R.A. (1958). On convection cells induced by surface tension. J. Fluid Mech..

[39] Goussis D.A., Kelly R.E. (1991). Surface wave and thermocapillary instabilities in a liquid film flow. J. Fluid Mech..

[40] Nepomnyashchy A.A., Velarde M.G., Colinet P. (2006). Interfacial Wave Theory of Pattern Formation.

[41] Sadiq I.M.R., Usha R., Joo S.W. (2010). Instabilities in a liquid film flow over an inclined heated porous substrate. Chem. Eng. Sci..

